# Comparative Evaluation of Commercial Bulk-Fill Resin-Based Composites: Flexural Properties, Roughness, Water Sorption and Solubility, and Color Stability

**DOI:** 10.3390/dj14020117

**Published:** 2026-02-14

**Authors:** Khalid S. Almulhim, Sarah M. Alghamdi, Raghad S. Alqahtani, Jood K. Alsahiem, Afnan O. Al-Zain, Mohammed M. Gad, Abdulrahman A. Balhaddad

**Affiliations:** 1 Department of Restorative Dental Sciences, College of Dentistry, Imam Abdulrahman Bin Faisal University, Dammam 31441, Saudi Arabia; ksalmulhim@iau.edu.sa; 2 College of Dentistry, Imam Abdulrahman Bin Faisal University, Dammam 31441, Saudi Arabia; 3 Department of Restorative Dentistry, Faculty of Dentistry, King Abdulaziz University, Jeddah 21589, Saudi Arabia; 4 Department of Substitutive Dental Sciences, College of Dentistry, Imam Abdulrahman Bin Faisal University, Dammam 31441, Saudi Arabia; mmjad@iau.edu.sa

**Keywords:** bulk-fill resin composites, flexural strength, color stability, surface roughness, mechanical properties

## Abstract

**Background/Objectives**: Bulk-fill (BF) resin-based composites (RBCs) have become increasingly popular due to their efficient placement. However, there is a lack of comprehensive performance comparisons among commercially available BF RBCs. In standardized curing conditions, this study aimed to compare the mechanical performance, water sorption and solubility, surface roughness, and color stability of commercially available BF RBCs with different consistencies (flowable and packable). **Methods**: Ten BF RBCs, along with a conventional RBC (control), were evaluated. Flexural strength and elastic modulus were measured using a three-point bending test. Water sorption and solubility were assessed after 28-day water storage. Color (Δ*E*_00_) and surface roughness (ΔRa) changes were measured after 28-day immersion in water, Pepsi, or coffee. One-way ANOVA and Tukey’s tests analyzed the data. **Results**: 3M Flow, Shofu Bulk, and Ivoclar Flow revealed lower strength (*p* < 0.001) compared to 3M Bulk (132.17 ± 12.54 MPa) and the control (124.56 ± 15.60 MPa). Shofu Bulk (24.68 ± 12.55 µg/mm^3^) and Ivoclar Flow (27.11 ± 6.27 µg/mm^3^) were the least affected by water sorption. While Shofu Bulk (13.98 ± 11.39 µg/mm^3^), Ivoclar Flow (20.28 ± 6.64 µg/mm^3^), and SDR (20.84 ± 9.74 µg/mm^3^) exhibited the lowest solubility (*p* < 0.01). After water and Pepsi immersion, FGM Bulk showed a significant color change compared to 3M Bulk and Ivoclar Bulk (*p* < 0.05). Following coffee immersion, Shofu Bulk (17.38 ± 1.82) revealed significant color changes (*p* < 0.001). Increased surface roughness was observed in 3M Bulk and Ivoclar Bulk after water immersion, Shofu Bulk after Pepsi immersion, and FGM Bulk after coffee immersion. **Conclusions**: BF RBCs exhibit notable variability in their intrinsic properties. 3M Bulk and Control showed the highest strength, while Shofu Bulk had significant color changes.

## 1. Introduction

Bulk-fill (BF) resin-based composites (RBCs) were developed to simplify restorative procedures by allowing placement in increments of up to 4–5 mm, thereby reducing clinical time and minimizing knit lines and the risk of void formation associated with incremental layering techniques [1,2]. Their enhanced translucency and modified photoinitiator systems are intended to promote more efficient light curing in thicker increments [3,4]. BF RBCs may also contain different photoinitiator systems that can modulate and optimize the polymerization of a thick RBC layer [5]. Moreover, recent BF RBCs can achieve a more cross-linking and enhanced depth of cure by undergoing reversible addition–fragmentation chain transfer [5].

There is an argument concerning the concept validity of the BF RBC systems, especially with the emergence of new commercial materials in the market. While most manufacturers claim that BF RBCs can be placed and cured up to a 4–5 mm increment, some studies found that the depth of cure might not be that efficient at the bottom layers of the BF RBCs [6,7]. In addition, it has been found that some BF RBCs were associated with lower mechanical-physical properties compared to the conventional incremental RBCs [8,9,10]. Therefore, it is important to test the mechanical and physical properties of these materials to validate their capabilities to withstand the harsh environment of the oral cavity.

Another clinical concern following restorative treatment is the exposure of dental restorations to different staining and acidic solutions inside the oral cavity. Frequent exposure to such solutions may affect the color and roughness of RBCs [11,12]. The change in the restorations’ color over time may compromise the esthetic appearance of such restorations [11,12]. In addition, topography changes such as increased surface roughness and reduced microhardness may degrade the material and accelerate plaque accumulation, which can lead to secondary caries [13,14]. Therefore, it is critical for any dental restoration to demonstrate excellent color stability and minimum topography changes when it is exposed to highly acidic solutions [11,12,13,14].

Previous comparative studies have typically focused on isolated properties (e.g., only mechanical characteristics or only color stability) or evaluated limited numbers of materials, making cross-study comparisons problematic due to methodological variations. This shortage of integrated assessments restricts clinicians’ ability to make informed, evidence-based decisions regarding material selection. To the authors’ knowledge, no previous study has simultaneously examined a broad range of BF RBCs with different consistencies under identical conditions while evaluating their performance across key domains, including mechanical properties, physical behavior, surface roughness, and color stability.

This study aimed to conduct a comprehensive comparative evaluation of the intrinsic properties of ten commercially available BF RBCs. This evaluation for the intrinsic properties was designed to be achieved under standardized curing conditions to assess their mechanical performance (flexural strength and elastic modulus), physical behavior (water sorption and solubility), as well as their color stability and surface topography before and after immersion in various staining solutions. The novelty of this investigation lies in three key aspects: (1) the simultaneous evaluation of multiple material consistencies (flowable and packable) under identical experimental conditions; (2) the integration of mechanical, physical, surface, and optical properties within a single comparative framework; and (3) the generation of a comprehensive performance profile that enables direct material comparison across clinically relevant parameters, facilitating evidence-based material selection. This integrated approach addresses a critical gap in the literature where previous investigations have examined these properties in isolation or under non-standardized conditions, thereby limiting the translational value of their findings for clinical practice. Therefore, this study hypothesized that the BF RBCs tested would exhibit similar flexural strength, elastic modulus, water sorption and solubility, and comparable roughness and color changes after immersion in beverage solutions when compared to the conventional incremental RBC used as a control.

## 2. Materials and Methods

### 2.1. Study Design and Sample Size Calculation

**Figure 1:**
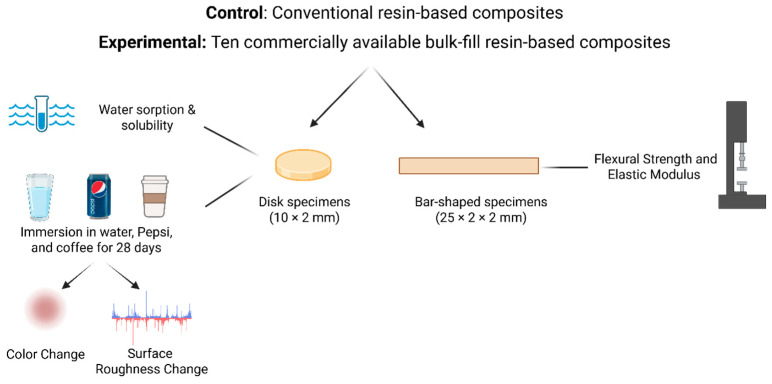
A schematic drawing showing the design of the study. Ten experimental bulk-fill resin-based composites and conventional incremental resin-based composites were examined for their flexural strength, elastic modulus, water sorption, and solubility. Additionally, roughness and color changes after immersion in coffee, tea, and distilled water for 28 days were assessed.

The list of materials is described in Table 1. Ten BF RBCs were used as experimental groups, while one commercial conventional incremental RBC was used as a control. Six samples per group were used for the flexural strength and elastic modulus, eight samples per group for water sorption and solubility, and ten samples for color and roughness changes. This sample size was used following previous investigations that found this number is enough to illustrate significant differences [15,16,17,18,19]. Following data collection and analysis, a post hoc power analysis was conducted to verify the adequacy of the selected sample size. The analysis confirmed that the chosen sample size provided sufficient statistical power (>80%) to detect significant differences among the tested materials. All specimens were cured at 0 mm distance from the sample top, and each RBC group was cured according to the manufacturer’s instructions using a multiple-emission-peak light-emitting diode (Valo Cordless, Ultradent, South Jordan, UT, USA) with a 10 mm internal diameter. The LCU power was approximately 800 mW, irradiance 1000 mW/cm^2^, and radiant exposure 10 J/cm^2^, and wavelength range 385–515 nm measured using the top sensor of the Managing Accurate Resin Curing- Light Corrector (MARC-LC, BlueLight Analytics Inc., Halifax, NS, Canada). The study design and the conducted tests are shown in Figure 1. Each test was conducted by a single calibrated operator for standardization purposes.

### 2.2. Flexural Strength and Elastic Modulus

A stainless-steel mold of 2 × 2 × 25 mm was used to fabricate the samples. The samples were cured using Valo Cordless with an irradiance of >1000 mW/cm^2^ in correspondence to the curing time stated in Table 1. Then, following the (ISO) 4049:2000(E) [20], the flexural strength and elastic modulus were measured via a three-point flexural test with a 10 mm span at a crosshead speed of 1 mm/min (Model LRX Plus, Ametek Instruments, Fareham, England) using the following equations:

$$Flexural\;strength\;(S)\;=\;3PL/{2bh}^{2}$$

*P* = fracture load, *L* = the span, *b* = specimen width, and *h* = specimen thickness.

$$Elastic\;modulus\;(E)=(P/d)(L^{3}/[{4bh}^{3}])$$

where *P* (the load) divided by *d* (displacement) is the slope in the linear elastic region.

### 2.3. Water Sorption and Water Solubility

The BF RBC samples were prepared with a diameter of 10 mm and a thickness of 2 mm with a mylar strip at the top and bottom of the samples before the curing procedure. The samples were cured using Valo Cordless with an irradiance of >1000 mW/cm^2^. Water sorption and solubility tests were performed in accordance with the (ISO) 4049:2009(E) [20]. To determine the volume (V) of each specimen, the average measurements of diameter and thickness were calculated. The diameter was measured at three different locations, and the average of these measurements was recorded. For thickness, five measurements were taken, with one at the center and the remaining four at the edges. After that, the volume of the samples was calculated.

The specimens were stored in a desiccator containing dried silica gel for 23 h at a temperature of 37 °C (±1 °C). Following this, they were transferred to a second desiccator and kept for an additional hour at 23 °C (±2 °C). The specimens were then weighed on a scale with an accuracy of 0.1 mg. This desiccation process continued until a stable mass (*m*1) was reached. The dried specimens were subsequently immersed in water for 28 days at 37 °C (±1 °C). After this immersion, the surface water over the specimens was blotted away until no visible moisture was observed, waved in the air for 15 s, and then weighed again (*m*2). The water sorption value (µg/mm^3^) was calculated using the formula:

$$Wsp=\frac{m2-m3}{v}$$

Once the sorption weights were recorded, the specimens were dried again to reach a constant mass (*m*3) using the desiccator. The solubility values (µg/mm^3^) of the specimens were then calculated using the formula:

$$Wsl=\frac{m1-m3}{v}$$

### 2.4. Color Stability and Topography Changes

The BF RBCs were prepared with a diameter of 10 mm and a thickness of 2 mm with a mylar strip at the top and bottom of the samples before the curing procedure. After curing with Valo Cordless, BF RBCs’ color and roughness were obtained at baseline and after immersion in different solutions (distilled water, Pepsi, and coffee) for 28 days. The Color-Eye 7000A digital spectrophotometer (X-rite, Grand Rapids, MI, USA), paired with SpectraMagic NX software (RM2002QC, Konica Minolta Corp., Ramsey, Japan), was employed to analyze color variations in the specimens by differentiating between transmitted and reflected light. This analysis relied on the CIE L*a*b* color space values. Each measurement was conducted three times, and the average values for the CIE L*a*b* data were computed [16]. The color difference was quantified using the CIED2000 equation:

$${\Delta E}_{00}\;=\;\sqrt{{\left(\frac{{∆L}^{\prime }}{K_{L}S_{L}}\right)}^{2}+{\left(\frac{{∆C^{\prime }}_{ab}}{K_{C}S_{C}}\right)}^{2}\;+{\left(\frac{{∆H^{\prime }}_{ab}}{K_{H}S_{H}}\right)}^{2}+R_{T}\left(\frac{{∆C^{\prime }}_{ab}}{K_{C}S_{C}}\right)\left(\frac{{∆H^{\prime }}_{ab}}{K_{H}S_{H}}\right)}$$

In this equation, ${\Delta E}_{00}$ indicates the extent of color change, while L*, a*, and b* are the color parameters involved in the measurement.

The average surface roughness change (∆Ra) measurements were assessed using a non-contact profilometer (Contour Gt-K1 optical profiler; Bruker Nano, Inc., Tucson, AZ, USA) as previously described [17]. To maintain measurement consistency, a pre-designed mold to direct the scanning process across different areas at the center of the specimens was used. Three random measurements were obtained at a speed of 0.5 mm per second. The measurements had a cutoff depth of 0.8 mm, scanned an area of 639 μm by 479 μm, featured a lateral resolution of 0.33 μm, a vertical resolution of less than 0.1 nm, and operated at a magnification of ×20 [21]. Each specimen was scanned at three distinct sites, and the corresponding average for Ra was obtained.

Once the baseline values were obtained, the samples were submerged in various solutions: (1) distilled water, (2) Pepsi (International Refreshments Company LTD, Riyadh, Saudi Arabia), and (3) coffee (NESCAFE, Girona, Spain). The coffee was prepared according to the manufacturer’s recommendations, and it was kept cooling for 5 min before the immersion. This immersion lasted for 28 days, simulating the equivalent of 2.5 years of clinical use [22,23]. The changes in color (Δ*E*_00_) and roughness (∆Ra) values before and after immersion were calculated by subtracting the values recorded after the immersion from those measured prior to it (the baseline).

### 2.5. Statistical Analysis

Descriptive statistics (mean, standard deviation, frequency, and percentages) were used to summarize the information. Sigma Plot 12.0; SYSTAT (Systat Software Inc. (SSI), San Jose, CA, USA) recorded and analyzed the data. Data normality and distribution were checked using the Shapiro–Wilk test. One-way ANOVA followed by post hoc Tukey test was used to compare the outcomes. This study used post-immersion Δ-values as the dependent variables for color and roughness. The study compared the tested BF RBCs separately for each immersion condition to directly address the primary research question. A *p*-value of < 0.05 was considered statistically significant.

## 3. Results

**Figure 2:**
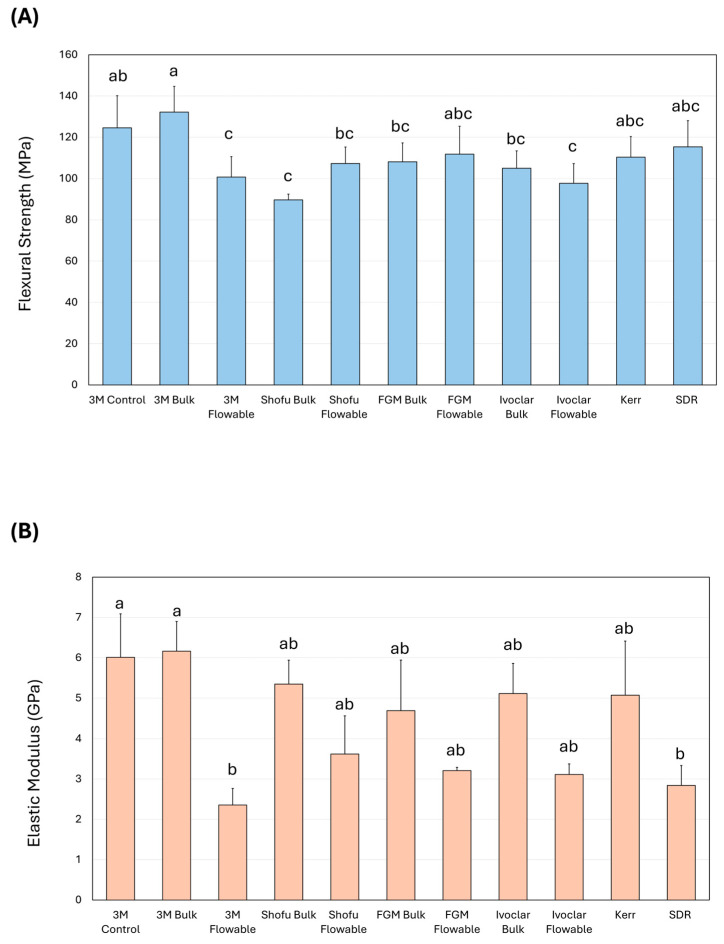
Assessment of the (**A**) the flexural strength and (**B**) elastic modulus values (mean ± SD) of the investigated bulk-fill resin-based composites. Different letters indicate a significant difference (*p* < 0.05).

The results of the study revealed significant variations in the mechanical and physical properties among the tested BF RBCs. The 3M Bulk (132.17 ± 12.54 MPa) and 3M Control (124.56 ± 15.60 MPa) groups exhibited the highest flexural strength (Figure 2A), which was significantly higher (*p* < 0.001) than 3M Flow, Shofu Bulk, Shofu Flow, FGM Bulk, Ivoclar Bulk, and Ivoclar Flow. Similarly, the elastic modulus values (Figure 2B) of 3M Bulk (6.01 ± 1.08 GPa) and 3M Control (6.16 ± 0.74 GPa) were the highest among the groups, and they were significantly higher than 3M Flow (2.35 ± 0.41 GPa) and SDR (2.84 ± 0.49 GPa).

**Figure 3:**
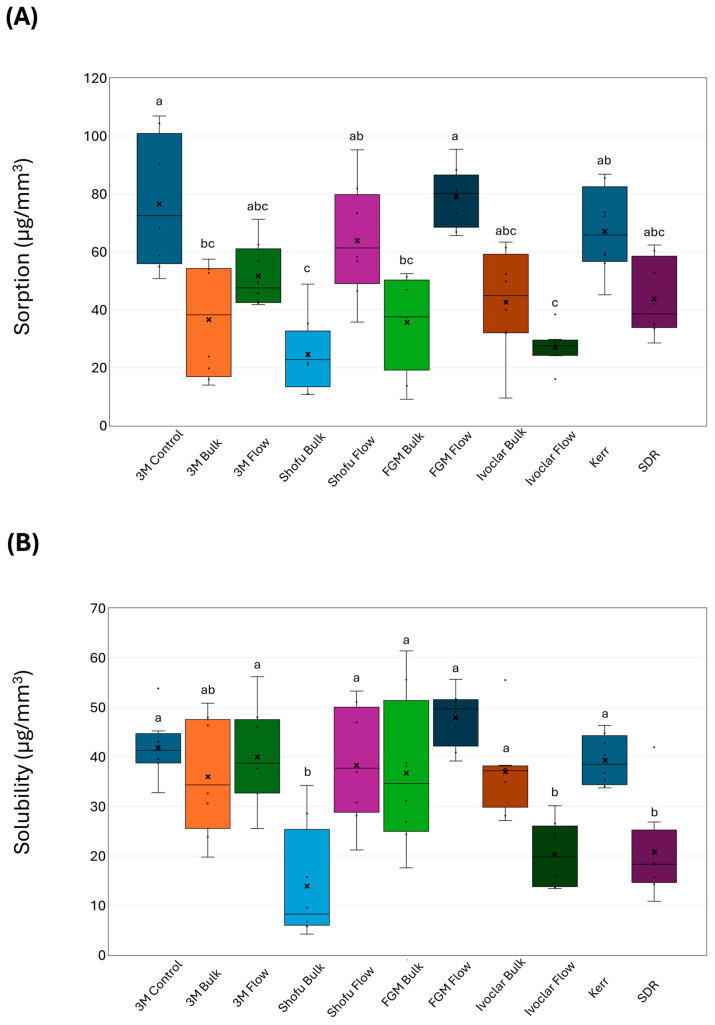
Assessment of the (**A**) water sorption and (**B**) solubility values (mean ± SD) of the investigated bulk-fill resin-based composites after 28 days of distilled water immersion. Different letters indicate a significant difference (*p* < 0.05).

For the water sorption experiment (Figure 3A), the least affected BF composites were Shofu Bulk (24.68 ± 12.55 µg/mm^3^) and Ivoclar Flow (27.11 ± 6.27 µg/mm^3^), and this was significant (*p* < 0.01) compared to 3M Control, Shofu Flow, FGM Flow, and Kerr. For the water solubility (Figure 3B), Shofu Bulk (13.98 ± 11.39 µg/mm^3^), Ivoclar Flow (20.28 ± 6.64 µg/mm^3^), and SDR (20.84 ± 9.74 µg/mm^3^) revealed significantly lower solubility (*p* < 0.01) compared to all the other groups except the 3M Bulk.

**Figure 4:**
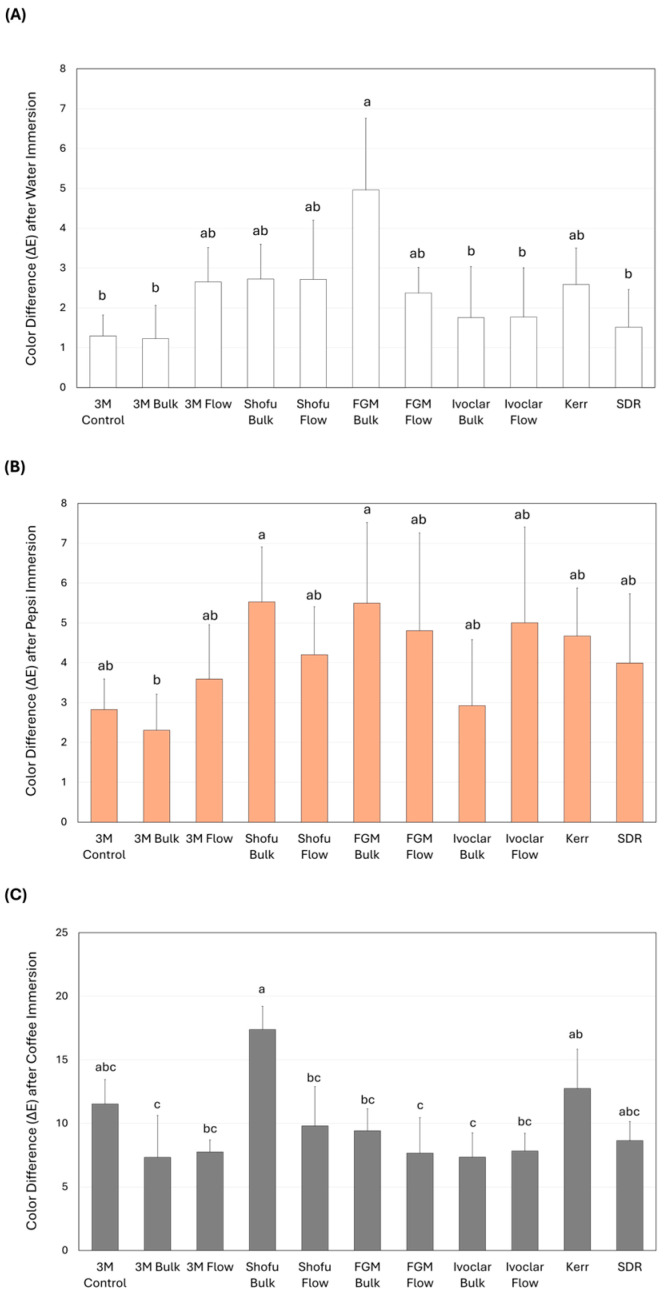
The color change (∆E) of the bulk-fill resin-based composites (mean ± SD) after 28 days of immersion. The color change was estimated after immersion in (**A**) distilled water, (**B**) Pepsi, and (**C**) coffee. Different letters indicate a significant difference (*p* < 0.05).

In terms of color change following water immersion (Figure 4A), FGM Bulk (4.96 ± 1.79 Δ*E*_00_) showed significant color change compared to several other materials, including 3M Control (1.30 ± 0.52 Δ*E*_00_), 3M Bulk (1.23 ± 0.83 Δ*E*_00_), Ivoclar Bulk (1.75 ± 1.28 Δ*E*_00_), Ivoclar Flow (1.24 ± 1.77 Δ*E*_00_), and SDR (1.51 ± 0.94 Δ*E*_00_) (*p* < 0.05). Following Pepsi immersion (Figure 4B), Shofu Bulk (5.53 ± 1.38 Δ*E*_00_) and FGM Bulk (5.50 ± 2.02 Δ*E*_00_) revealed the highest color change, which was significant (*p* < 0.05) compared to the 3M Bulk (2.31 ± 0.90 Δ*E*_00_). In Figure 4C, the Shofu Bulk revealed the highest amount of color change (17.38 ± 1.82 Δ*E*_00_) after coffee immersion, which was significantly (*p* < 0.05) higher than all the BF RBCs except the Kerr and SDR.

**Figure 5:**
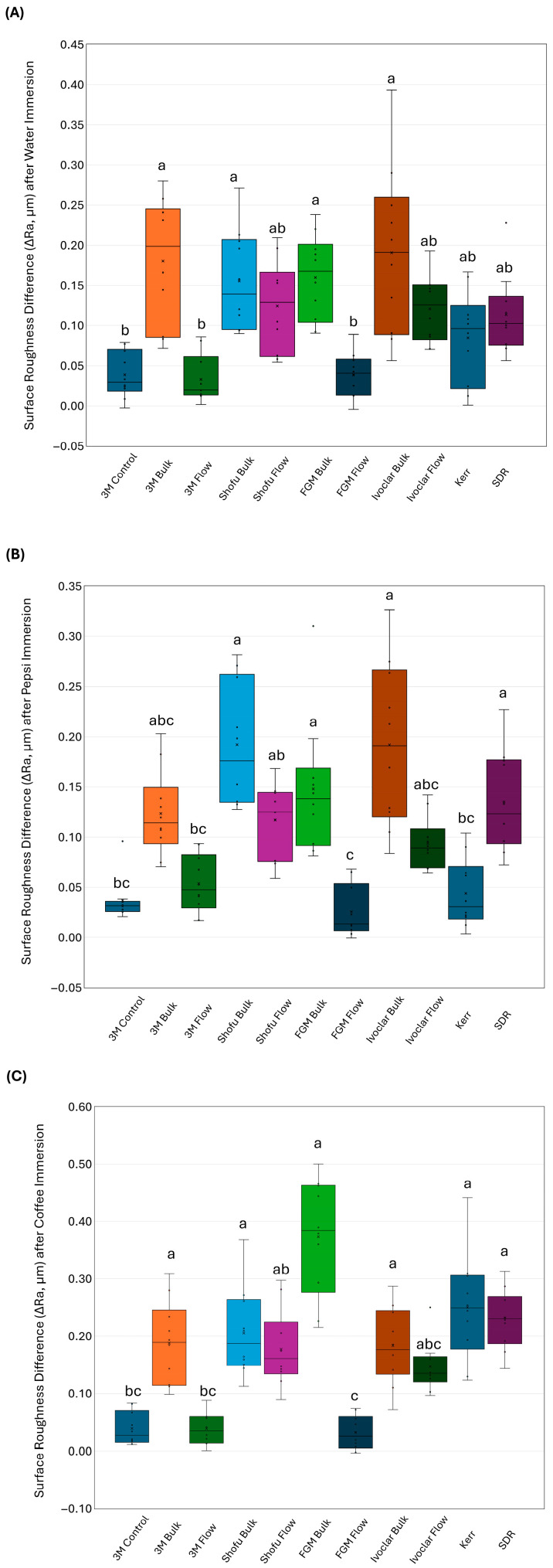
The average surface roughness change (∆Ra) of the bulk-fill composites (mean ± SD) after 28 days of immersion. The roughness change was estimated after immersion in (**A**) distilled water, (**B**) Pepsi, and (**C**) coffee. Different letters indicate a significant difference (*p* < 0.05).

Figure 5 shows the results of the surface roughness change (∆Ra). 3M Bulk (0.18 ± 0.08 µm) and Ivoclar Bulk (0.19 ± 0.10 µm) showed increased roughness after water exposure (Figure 5A), which was significantly (*p* < 0.01) higher than 3M Control (0.04 ± 0.02 µm), 3M Flow (0.03 ± 0.03 µm), and FGM Flow (0.04 ± 0.04 µm). After Pepsi immersion (Figure 5B), Shofu Bulk (0.19 ± 0.06 µm) and Ivoclar Bulk (0.19 ± 0.08 µm) revealed higher roughness change compared to the 3M Control (0.04 ± 0.02 µm), 3M Flow (0.05 ± 0.03 µm), FGM Flow (0.02 ± 0.02 µm), and Kerr (0.04 ± 0.03 µm). While after coffee immersion (Figure 5C), the lowest amount of roughness change was observed in the 3M Control (0.04 ± 0.03 µm), 3M Flow (0.04 ± 0.03 µm), and FGM Flow (0.03 ± 0.03 µm), which was significantly less (*p* < 0.05) compared to 3M Bulk, Shofu Bulk, FGM Flow, Ivoclar Bulk, Kerr, and SDR.

## 4. Discussion

In this study, we examined the mechanical and physical properties of ten brands of BF RBCs. These brands have been included for their popularity in the market, as many clinicians use them in dental practice. The brands investigated in this study displayed significant variations in strength and surface roughness, suggesting that the choice of brand could be tailored to specific clinical applications. Based on the findings of the present study, showing variation between brands in terms of tested properties compared to the control, the study hypothesis was rejected. In this study, the comparison between the brands tested was limited by a lack of information in the manufacturers’ manuals. Key details regarding the resin matrix formulation, types of photoinitiators, and the type, size, and loading of fillers were absent. Consequently, this information gap hindered a thorough comparison of the BF RBCs in terms of their mechanical and physical performance. A multi-peak light-curing unit was employed to accommodate the potential presence of alternative photoinitiators. Photoinitiators that are activated in the violet wavelength range would not be adequately triggered if a single-emission peak device were used, leading to insufficient polymerization and compromised mechanical and physical properties. To mitigate this risk, a multi-emission LED device was utilized to ensure the proper activation of all photoinitiators.

Both flowable and packable BF RBCs were included in our investigation. Flowable BF RBCs, known for their low viscosity, easily adapt to cavity shapes, making them ideal for precise applications. They can be placed in bulk, which reduces the time required for layering and curing, and they offer flexibility along with good optical properties that match the color of natural teeth [24]. The packable BF RBCs possess a higher viscosity, providing the necessary strength for load-bearing areas in the mouth. They are also suitable for bulk placement, which streamlines the application process, although they often require specific curing protocols due to their density. With superior mechanical properties, packable RBCs are expected to demonstrate greater compressive strength and wear resistance compared to their flowable counterparts. However, many dental clinicians use both materials in both low-stress and high-stress areas, despite their respective strengths and filler load characteristics. Therefore, assessing the strength of this class of materials and comparing the two viscosity types is essential. Our results revealed that some flowable BF RBCs were stronger than packable ones, but it is essential to highlight that the outcomes were obtained immediately after specimen fabrication, and different strength behaviors could be observed following artificial aging or in clinical settings. Furthermore, the study findings revealed that only the 3M Bulk surpassed the conventional control RBC in terms of flexural strength, highlighting its superiority over the other BF RBCs. While Table 1 shows the chemical composition of the tested materials, it is difficult to speculate on the rationale for the strength variation based on the composition. This is mainly due to the fact that the exact composition by percentage is not shown, and the filler load is not mentioned in most of the formulations. For instance, the 3M Bulk and Shofu Bulk have almost the same chemical composition, but there was a big variation in their flexural strength.

It is worth mentioning that all the tested BF RBCs exhibited a flexural strength exceeding the critical threshold of 80 MPa, as stipulated by ISO standards. This finding is significant because it validates the capability of these materials to withstand the functional stresses encountered in clinical settings. Flexural strength is a crucial mechanical property for dental RBCs, as it directly relates to their ability to endure occlusal forces during chewing, which can be substantial, especially in posterior teeth [15]. Therefore, while significant differences were observed among the various BF composite brands, all tested materials exceeded the ISO 4049 requirement of 80 MPa minimum flexural strength for resin-based composites. However, it is essential to note that the results reported are based on measurements taken immediately after sample preparation. This timing is critical, as it does not account for potential changes in material properties over time due to factors such as moisture absorption, enzymes, pH fluctuations, mechanical stress, or degradation. Aging and moisture absorption can diffuse through the resin matrix, causing swelling and plasticization. This process leads to hydrolytic degradation of ester linkages, which is detrimental to the mechanical and physical properties of RBCs [25,26]. Therefore, testing the BF composites after actual or artificial aging would provide a more comprehensive understanding of their long-term performance and durability.

Water sorption and solubility are fundamental factors influencing the long-term mechanical integrity and esthetic stability of resin composites [27], including bulk-fill materials. Excess uptake of moisture can expand the gap between the polymer chains, depending on the density of the cross-linking structure [28]. This could lead to softening of the organic matrix, promote hydrolytic degradation, and increase the release of residual components, while solubility contributes to the gradual weakening of the material [29]. Bulk-fill composites, placed in thicker increments, may display different patterns of sorption and solubility depending on their resin chemistry, filler content, and the efficiency of polymerization. These behaviors also affect color stability, since greater water absorption facilitates pigment penetration and chemical changes over time [30].

In our current study, the results of water sorption and solubility agree with findings reported previously [28], where conventional RBC showed higher sorption levels than most BF materials. This study demonstrated that Shofu bulk had the lowest water solubility and sorption, whereas FGM Flow exhibited the highest values. Additionally, there was no difference in solubility and sorption between flowable and packable BF RBCs, as variations were observed between brands despite their viscosity. It is important to note that water uptake is a multifactorial process influenced by several material characteristics rather than solely by the storage medium. Moreover, the tendency of RBCs to absorb water is strongly linked to their formulation: higher filler content and a less hydrophilic resin matrix generally reduce sorption, with resin systems based on BisEMA or UDMA–BisEMA demonstrating greater resistance to moisture compared with more hydrophilic BisGMA-rich matrices [31]. Accordingly, due to variations in resin matrix composition and filler content, the RBCs in our study showed corresponding differences in water sorption and solubility outcomes.

In this study, the solubility of the RBCs exhibited considerable variation and did not consistently correlate with filler content, although filler loading remains a significant determinant of water sorption. Solubility is a multifactorial property, influenced not only by the resin matrix and filler characteristics but also by the amount of unreacted residual monomers released from the polymer network [32]. The degree of polymerization critically governs the quantity of these leachable monomers, while their molecular size further affects their diffusion and degradation, with smaller molecules exhibiting higher mobility and faster release [33]. Therefore, the observed differences in solubility among the tested RBCs can be attributed to variations in resin composition, monomer type, and polymerization efficiency, highlighting the complex interplay of material-specific factors in determining solubility behavior. Assessing solubility and sorption beyond 28 days may also provide greater insight into the long-term performance of BF RBCs and their resistance to aging degradation.

Color stability of RBC materials is an important characteristic for the esthetic survival of dental restorations, as it directly affects both aesthetics and patient satisfaction [34]. Over time, RBCs are exposed to various factors such as food, beverages, and environmental conditions that can lead to discoloration or staining. If a restoration loses its original color, it may become noticeable and detract from the natural appearance of the surrounding teeth, potentially leading to dissatisfaction and the need for replacement [35]. The color stability of RBCs is influenced by both the resin matrix composition and the characteristics of filler particles, including their type and size [36,37]. In particular, the resin matrix plays a crucial role in determining whether the RBC will undergo discoloration. Research has demonstrated that various properties of resin composition, including the chemical structure of resin monomers, the concentration and types of activators, initiators, and inhibitors, as well as the oxidation of unreacted monomers, contribute to the susceptibility of RBCs to discoloration [36,37].

Color stability is indicative of the material’s durability; RBCs that maintain their color are often more resistant to degradation and wear. This resistance contributes to the overall longevity of the restoration, ensuring it remains functional and visually appealing throughout its lifespan. Therefore, selecting RBCs with high color stability is essential for achieving lasting and aesthetically pleasing dental restorations. It is well known that when the color difference (∆E00) surpasses 3.5, the change in color becomes apparent in a clinical setting and is deemed unacceptable for use [16]. In this investigation, following water immersion, only the FGM bulk exceeded this critical threshold. While following the Pepsi immersion, all the brands exceeded the value of 3.5, except the control, 3M Bulk, and Ivoclar Flow. Finally, all the brands were associated with a color change of more than 3.5 following coffee immersion. It is important to note that whether the observed color change is superficial or affects the intrinsic body of the RBC remains unknown. Typically, discoloration begins superficially, but with continued exposure to staining agents, pigmentation may diffuse throughout the restoration body, complicating clinical management of the discoloration.

Most of the BF RBCs tested here revealed greater color change than the conventional RBC following water and Pepsi immersion. Several factors may account for the increased pigmentation observed in BF RBCs, including changes to monomer composition, elevated translucency, and the integration of pre-polymerized particles into the resin matrix [38]. However, after coffee immersion, the color change observed in both BF and conventional RBCs was similar, largely due to the pronounced discoloration effect of coffee, which superseded the role of RBC chemical composition in determining color stability. 3M Bulk was associated with less color change compared to the control in all the tested solutions, suggesting its capability to resist staining due to aging. In contrast, Shofu Bulk and Kerr were associated with more color change compared to the control in all the solutions as well.

The roughness of RBC materials plays a critical role in the longevity and survival of dental restorations [23]. A smooth surface is essential for minimizing plaque accumulation and reducing the risk of secondary caries, as rough surfaces can harbor bacteria and facilitate biofilm formation [39]. Furthermore, the surface roughness influences the wear resistance of the restoration; smoother RBCs tend to withstand occlusal forces better and are less prone to chipping or degradation over time [40]. Additionally, the aesthetic properties of the restoration are affected by surface texture; a well-finished, smooth surface enhances the optical qualities of the composite, contributing to a more natural appearance [40]. Therefore, achieving the appropriate roughness of the surface during the finishing and polishing stages is vital not only for the functional performance of the restoration but also for its overall success and patient satisfaction. The results of this study revealed that only 3M flow and FGM flow roughness were comparable to the control, suggesting that these two brands may be more resistant to aging and degradation; thus, their clinical longevity may be better than that of other BF RBCs.

Among the different types of fillers used in dentistry, surface pre-reacted glass-ionomer (S-PRG) fillers have gained attention recently. S-PRG in the resin formulation represents a multifunctional approach to RBCs enhancement, conferring antibacterial activity, ion-exchange capacity, acid-buffering properties, remineralization potential, and improved esthetics [41]. In Shofu BF RBCs, this biomimetic filler system releases therapeutic ions, including fluoride, strontium, borate, and silicate, while maintaining the capacity for fluoride recharge, characteristics that position these materials as bioactive therapeutic agents rather than conventional inert restoratives [41]. Despite these biological advantages, the present investigation revealed compromised physical performance of Shofu BF RBCs, manifesting as elevated surface roughness values and heightened susceptibility to color alterations following immersion in staining media. This apparent discrepancy suggests that the incorporation of S-PRG particles, while enhancing bioactivity and remineralization potential, may inadvertently compromise surface integrity and color stability when the ions are released from the resin matrix.

While laboratory testing provides essential insights into material properties, clinical performance represents the ultimate benchmark for restorative materials. Recent systematic reviews support the clinical viability of bulk-fill composites in both primary and permanent dentition. In primary teeth, Sarapultseva et al. demonstrated that bulk-fill composites achieve retention and survival rates comparable to incremental placement techniques, with the added benefit of reduced operative time [42]. Similarly, in permanent posterior teeth, Abreu et al. reported in a systematic review and meta-analysis that bulk-fill resin composites provide survival and clinical performance broadly equivalent to conventional incremental composites while simplifying the placement procedure [43]. These clinical findings suggest that the mechanical properties of BF RBCs may translate to acceptable clinical outcomes. However, it is important to recognize that laboratory studies, including this one, represent controlled conditions that do not fully replicate the complex oral environment. Therefore, this study’s findings should be considered supportive evidence that complements, rather than definitively predicts, clinical performance.

In summary, this study offers valuable insights into the mechanical and physical performance, as well as the surface roughness, of BF RBCs. The 3M Bulk and 3M Control exhibited the highest flexural strengths and elastic modulus values. Shofu Bulk and Ivoclar Flow demonstrated the least water sorption, significantly lower compared to 3M Control. For water solubility, Shofu Bulk, Ivoclar Flow, and SDR exhibited lower solubility. Following water immersion, FGM Bulk exhibited a significant change in color compared to other materials. After Pepsi immersion, Shofu Bulk and FGM Bulk showed the greatest color changes. In coffee immersion, Shofu Bulk experienced the most significant color change, notably higher than all BF RBCs except for Kerr and SDR. For surface roughness evaluation, 3M Bulk and Ivoclar Bulk exhibited increased roughness. After the Pepsi immersion, Shofu Bulk and Ivoclar Bulk had higher roughness changes compared to the 3M Control and others. Finally, the least change in roughness after coffee immersion was observed in 3M Control, 3M Flow, and FGM Flow.

Several limitations must be acknowledged to provide a clearer context for the findings. Firstly, this research was conducted in vitro under conditions that do not fully replicate the complexities of long-term clinical service. This study assessed the intrinsic properties of the BF RBCs utilizing specimens with 2 mm thickness to exclude the impact of depth-related polymerization on the tested properties. As a result, the findings should not be extrapolated to determine bulk-increment safety or clinical curing adequacy. In addition, a significant limitation is the absence of aging protocols prior to mechanical testing; specimens were not subjected to thermocycling or prolonged water storage that would simulate the thermal and hydrolytic stresses encountered during extended intraoral function. Additionally, factors such as saliva, enzymatic degradation, masticatory forces, and bacterial biofilm formation, which can significantly influence material behavior and degradation over time in vivo, were not accounted for in this study. The mechanical properties reported, therefore, represent initial material performance rather than aged material behavior, which may differ substantially after months or years of clinical service. Additionally, while this study focused on specific properties of BF RBCs, several other crucial mechanical and physical characteristics were not evaluated in this investigation. Furthermore, future investigations should consider expanding the range of BF RBCs tested, including various manufacturers and formulations, to provide a more representative overview of material behavior across different products. Lastly, to further validate the performance and longevity of these restorations, future studies would greatly benefit from conducting clinical trials. Such trials could assess how these materials perform over extended periods in actual oral conditions, providing insights that are crucial for clinical applicability.

## 5. Conclusions

When their intrinsic properties were assessed under standardized curing conditions, bulk-fill resin-based composites exhibited significant property variations. 3M Bulk and 3M Control demonstrated superior mechanical properties. Shofu Bulk and Ivoclar Flow showed the lowest water sorption/solubility. Coffee caused the greatest color changes, particularly in Shofu Bulk. Surface roughness increased most in Shofu and Ivoclar Bulk materials. These outcomes underscore the importance of material-specific selection to optimize clinical performance. Future studies with extended aging protocols are recommended. In addition, clinical trials are needed to validate the use of these brands of BF resin composites.

## Figures and Tables

**Table 1:** List of materials included in the study.

#	Material Brand Name	Chemical Composition	Curing Instructions
Control	Filtek Z350 XT (3M ESPE Dental Products, St. Paul, MN, USA)	Resins: Bis-GMA, UDMA, Bis-EMA, TEGDMA Fillers: Silica, zirconia, zirconia/silica nanoclusters	≥1000 mW/cm^2^ = 20 s ~600–800 mW/cm^2^ = 30 s
1	3M™ Filtek™ One Bulk Fill Restorative (3M ESPE Dental Products, St. Paul, MN, USA)	Resins: Bis-GMA, UDMA, Bis-MPEPP, TEGDMA, S-PRG Fillers: Fluoroboroaluminosilicate glass, Polymerization initiator, Pigments and others	Halogen = 20 s LED = 10 s
2	3M™ Filtek™ Bulk Fill Flowable Restorative (3M ESPE Dental Products, St. Paul, MN, USA)	Resins: Bis-GMA, TEGDMA, Bis-EMA, and Procrylat resins. Fillers: A combination of ytterbium trifluoride fillers with a range of particle sizes from 0.1 to 5.0 microns and zirconia/silica with a particle size range of 0.01 to 3.5 pm. The inorganic filler loading is approximately 64.5% by weight (42.5% by volume).	LED lights (with output 1000–2000 mW/cm^2^) All halogen lights/LED lights (with output 550–1000 mW/cm^2^)
3	Shofu Beautifil Bulk Restorative Composite (Shofu INC, Kyoto, Japan)	Resins: Bis-GMA, UDMA, Bis-MPEPP, TEGDMA, S-PRG Fillers: Fluoroboroaluminosilicate glass, Polymerization initiator, Pigments and others	Halogen light (Light intensity: ≥500 mW/cm^2^) = 20 s LED light (Light intensity: ≥1000 mW/cm^2^) = 10 s
4	Shofu Beautifil-Bulk Flowable (Shofu INC, Kyoto, Japan)	Resins: Bis-GMA, UDMA, Bis-MPEPP, TEGDMA, S-PRG Fillers: Fuoroboroaluminosilicate glass, Polymerization initiator, pigments and others	universal halogen = 20 s universal LED = 10 s Dentin halogen = 40 s Dentin LED = 20 s
5	OPUS Bulk-Fill APS (FGM, Lauderdale, FL, USA)	Resins: UDMA monomers, stabilizers, photoinitiating composition (APS) and co-initiators. Fillers: Silanized silicon dioxide (silica); stabilizers and pigments.	450–1000 mW/cm^2^ = 40 s 1000–2000 mW/cm^2^ =30 s
6	OPUS Bulk-Fill Flow APS (FGM, Lauderdale, FL, USA)	Resins: UDMA monomers, stabilizers, photoinitiating composition (APS) and coinitiator. Fillers: Inorganic load filler of silanized silica dioxide, stabilizers and pigments. Filler loading is approximately 68% by weight	20 s (With minimum power of 450 mW/cm^2^)
7	Tetric EvoCeram Bulk Fill (Ivoclar Vivadent, Schaan, Liechtenstein)	Resins: Dimethacrylates (19–21% weight). Fillers: Barium glass, prepolymer, ytterbium trifluoride and mixed oxide. Additives, catalysts, stabilizers and pigments are additional contents (<1.0% weight). The total content of inorganic fillers is 75–77% weight or 53–55% volume.	≥ 500 mW/cm^2^ = 20 s ≥1000 mw/cm^2^ = 10 s
8	Tetric EvoFlow Bulk Fill (Ivoclar Vivadent, Schaan, Liechtenstein)	Resins: Monomethacrylates and dimethacrylates (28 wt%). Fillers: Barium glass, ytterbium trifluoride and copolymers (71 wt%). Additives, initiators, stabilizers and pigments are additional ingredients (<1.0 wt%). The total content of inorganic fillers is 68.2 wt%/46.4 vol%. The particle size of the inorganic fillers ranges between 0.1 μm and 30 µm with a mean particle size of 5 μm	≥500 mW/cm^2^ = 20 s ≥1000 mw/cm^2^ = 10 s
9	SonicFill 3 Single-Fill (Kerr Corp, Orange, CA, USA)	Resins: Bis-EMA, Bis-GMA, and TEGDMA resins. Fillers: mixed oxides, barium glass filler, silica, and ytterbium trifluoride (about 81.5% by weight or 65.9% by volume). All shades have a primary particle size range of 40 nm to 10 microns. The inorganic filler is up to 75% by weight or 55% by volume.	light (with output 21,000 mW/cm^2^): 10 s
10	SDR flow+ Bulk Fill Flowable (Dentsply Sirona, Ballaigues, Switzerland)	Resins: High-MW modified UDMA with polymerization-modulator, plus TEGDMA, EBPADMA Fillers: Barium/strontium alumino-fluoro silicate glass, ytterbium fluoride	20 s with a curing light ≥ 1000 mW/cm^2^

Bis-MPEPP = poly-ethoxylated-bis-phenol-A-dimethacrylates; Bis-GMA = bisphenol A-glycidyl methacrylate; TEGDMA = Triethylene glycol dimethacrylate, UDMA = Urethane Dimethacrylate; PEGDMA = Poly(ethylene-glycol)-Dimethacrylate; Bis-EMA = 2,2-Bis [4-(2-methacryloxyethoxy)phenyl] propane; S-PRG = Surface Pre-Reacted Glass-ionomer.

## Data Availability

The original contributions presented in this study are included in the article. Further inquiries can be directed to the corresponding author.
